# First German-speaking harm reduction conference in Vienna

**DOI:** 10.1186/s12954-024-00932-z

**Published:** 2024-01-16

**Authors:** Larissa Steimle, Simon Fleißner, Hans Haltmayer, Thilo Beck, Alfred Springer, Heino Stöver

**Affiliations:** 1 Institute of Addiction Research, Frankfurt University of Applied Sciences, Frankfurt, Germany; 2 Suchthilfe Wien, Vienna, Austria; 3 Arud Centre for Addiction Medicine, Zurich, Switzerland; 4 Medical University of Vienna, Vienna, Austria

**Keywords:** Harm reduction, Tobacco, Harm reduction conference, Drug control policy

## Abstract

The first Harm Reduction DACH Conference [DACH = D (Germany), A (Austria), CH (Switzerland)] took place in Vienna on June 23rd, 2023, and focused on tobacco harm reduction. It is the first conference bringing together various experts of all three German-speaking countries to shed light on the subject of destigmatization and tobacco harm reduction and to share their experiences with the audience. All in all, the first German-speaking harm reduction conference has the goal to discuss and expand harm reduction in the German-speaking countries. This meeting report gives a brief overview of the conference.

## Background

The concept of harm reduction was developed in the 1980s, especially with regard to services for users of illegal substances, as an alternative to an exclusively abstinence-oriented drug aid. Although the concept of harm reduction for illegal drugs has found its way into many national drug strategies, the situation is somewhat different for legal drugs [1]. Even if the implementation of harm reduction measures in the German-speaking countries is still expandable in the case of illegal substances, it appears that so far there has been an insufficient transfer of the concept to legal substances in German-speaking countries, and that well-proven strategies of harm reduction have not yet been implemented in the field of legal substance use. In the German-speaking countries, there are a variety of organizations promoting and practicing harm reduction, but there is still no structure for a continuous exchange. Therefore, the aim of this conference was to bundle the expertise and activities in the various areas at the level of the professional societies and to jointly establish a conference that positions topics such as harm reduction, risk minimization and destigmatization, more strongly as a crosscutting issue and provides a format for exchange of experiences and opinions encompassing the whole range of use of legal and illegal substances and non-substance-related addictive disorders.

## First German-speaking harm reduction conference in Vienna

The first Harm Reduction DACH Conference (DACH = D (Germany), A (Austria), CH (Switzerland)) on June 23rd, 2023, in Vienna focused on tobacco harm reduction. Smoking of tobacco can cause a range of illnesses, such as lung cancer, COPD and strokes, and is responsible for 700.000 deaths each year in the European Union. Half of the people smoking die prematurely, which translates into an average loss of 14 years per smoker. In the European Union, smoking is the leading cause of preventable cancer, with 27% of all cancers being caused by tobacco smoking. Therefore, “tobacco consumption remains the largest avoidable health risk in the European Union” [2]. For this reason, the European Union and its Member States have been working to reduce the smoking of tobacco. However, according to the Tobacco Control Scale, a scale of 37 European countries quantifying the implementation of tobacco control policies, Austria (26), Germany (34) and Switzerland (36) are ranked within the last quarter of the scale [3]. In Austria, the proportion of the population that smokes daily is 20.6% [4] (2019), in Germany, 32.4% [5] (2023) of the population smokes, and in Switzerland, 27.1% [6] (2017). In comparison—in the UK, which ranks second on the Tobacco Control Scale, 13.3% [7] (2021) are still smoking. Therefore, tobacco control remains a highly relevant topic, especially in German-speaking countries. Particularly in relation to tobacco, harm reduction strategies are applied only very hesitantly and are often not part of the national health programs. For this reason, the conference brought various experts together to shed light on the subject of destigmatisation and tobacco harm reduction and to share their experiences with the audience.

The first keynote “Historical introduction to the concepts of harm reduction in the legal and illegal spheres” by Alfred Springer was followed by Bernhard Rupp’s keynote “Opportunities, risks and limitations of the harm reduction approach from an ethical perspective”. These two keynotes covered the basic topics and ideas about harm reduction focused on Austria, Switzerland and Germany. Then Heino Stöver talked about the “Application of harm reduction measures in tobacco addiction in practice”. Karl Erik Lund presented “The Scandinavian Experience of Tobacco Harm Reduction”, emphasizing the role snus had played in reducing smoking prevalence in the Scandinavian countries.

The next session included three parallel interactive workshops. In the workshop “Smoking: There is another way than ‘Quit or Die’" by Wolfgang Popp, the concrete realization of harm reduction strategies in the treatment of people who are unwilling or unable to quit smoking as an alternative to treatment with the goal of abstinence was discussed. Tobacco control policy in Germany was discussed in the workshop by Heino Stöver and Tobias Rüther on “Quitting Smoking with Alternative Approaches”. In particular, damage-reducing measures with regard to the possible legalization of cannabis in Germany and the problematic sale of nicotine pouches to minors in Austria. In the third workshop “The Differences in Harm Reduction in German-speaking Countries” by Alfred Uhl, the role of harm reduction in the national addiction strategies of Switzerland and Austria was presented and discussed.

After the presentations and during the discussions, there were no major differences of opinion on specific topics. The discussions mainly focused on the different policies in the three countries and served above all to exchange information about the respective situations. The different regulations of nicotine pouches with regard to the lack of youth protection in Austria, which not all participants were aware of, are a good example of the exchange of knowledge and experiences with the existing regulations.

## Conclusion

Overall, the conference highlighted that many tobacco harm reduction strategies are still not implemented in German-speaking countries, and there was a consensus that Germany, Switzerland and Austria have major deficits with regard to their tobacco control policies. While e-cigarettes, tobacco heaters, nicotine pouches and snus are considered to be less harmful [8, 9] and are therefore considered to be a good harm reduction option, especially for people with a history of multiple attempts to quit, the national health departments continue to ignore these facts. Overall, there is a need for a tobacco policy that include both abstinence-focused and evidence-based harm reduction strategies. Individualized strategies should support people who smoke to stop smoking to hasten reduction in smoking-related deaths. Particular attention must be paid to young people, who need protection to be protected from any kind of addiction. Countries such as Ireland, the UK and the Scandinavian countries can serve as a positive example. The second German-speaking Harm Reduction Conference will take place next year in Switzerland to continue the exchange and discussion about harm reduction in Germany, Austria and Switzerland and to promote good practice and evidence-based drug policy.
